# Dynamic Regulation of the Adenosine Kinase Gene during Early Postnatal Brain Development and Maturation

**DOI:** 10.3389/fnmol.2016.00099

**Published:** 2016-10-20

**Authors:** Katharina Kiese, Janos Jablonski, Detlev Boison, Katja Kobow

**Affiliations:** ^1^Department of Neuropathology, University Hospital ErlangenErlangen, Germany; ^2^Robert Stone Dow Neurobiology Laboratories, Legacy Research InstitutePortland, OR, USA

**Keywords:** adenosine kinase, specificity protein 1, rat, brain, development, epigenetic

## Abstract

The ubiquitous metabolic intermediary and nucleoside adenosine is a “master regulator” in all living systems. Under baseline conditions adenosine kinase (ADK) is the primary enzyme for the metabolic clearance of adenosine. By regulating the availability of adenosine, ADK is a critical upstream regulator of complex homeostatic and metabolic networks. Not surprisingly, ADK dysfunction is involved in several pathologies, including diabetes, epilepsy, and cancer. ADK protein exists in the two isoforms nuclear ADK-L, and cytoplasmic ADK-S, which are subject to dynamic expression changes during brain development and in response to brain injury; however, gene expression changes of the *Adk* gene as well as regulatory mechanisms that direct the cell-type and isoform specific expression of ADK have never been investigated. Here we analyzed potential gene regulatory mechanisms that may influence *Adk* expression including DNA promoter methylation, histone modifications and transcription factor binding. Our data suggest binding of transcription factor SP1 to the *Adk* promoter influences the regulation of *Adk* expression.

## Introduction

Adenosine kinase (ADK; EC 2.7.1.20) is an evolutionarily conserved phosphotransferase converting the purine ribonucleoside adenosine into 5′-adenosine-monophosphate (AMP) (Park and Gupta, 2008). This enzymatic reaction plays a fundamental role in determining the tissue tone of adenosine, which fulfills essential functions as a homeostatic and metabolic regulator in all living systems (Boison, 2013). ADK is the main enzyme in adenosine metabolism under baseline conditions and crucial to maintain adenosine homeostasis. ADK activity keeps adenosine levels low. Slight changes in enzyme activity result in major changes of adenosine concentration. Thereby, ADK has key regulatory functions in different cellular processes: As a central sensor and regulator of energy homeostasis its enzymatic activity is regulated by AMP, ADP, and ATP. Adenosine plays a key role in enzyme catalysis as part of the coenzymes nicotinamide adenine dinucleotide (NAD) and flavin adenine dinucleotide (FAD) (Denessiouk et al., 2001). It impacts intracellular signaling through four different types of adenosine receptors, which combine to regulate the second messenger cAMP (Fredholm et al., 2000). In the nucleus, ADK is a core element of nucleic acid metabolism and a regulator of cell proliferation (Ethier and Dobson, 1997; MacLaughlin et al., 1997). It is further an important regulator of epigenetic processes through its link to the transmethylation pathway (Boison et al., 2002b; Kobow and Blumcke, 2012; Williams-Karnesky et al., 2013). Therefore, tight regulation of ADK expression and activity becomes a necessity and it is not surprising that several pathologies such as epilepsy, diabetes or cancer are linked to ADK dysfunction (Pawelczyk et al., 2000; Saitoh et al., 2004; Duarte et al., 2006; Sakowicz-Burkiewicz et al., 2006; Giglioni et al., 2008; Li H. et al., 2008; Aronica et al., 2011; Masino et al., 2011; Tsuchiya et al., 2012).

During early postnatal rodent brain development Adk protein expression undergoes a coordinated developmental shift from a predominantly neuronal to a predominantly astrocytic expression pattern (Fedele et al., 2005; Studer et al., 2006). Adk exists in two isoforms: The long nuclear isoform Adk-L may play a specific role during developmental processes in the brain, presumably as epigenetic regulator, whereby Adk-L drives the flux of methyl-groups through the DNA transmethylation pathway. In contrast, the short cytoplasmic isoform Adk-S regulates the extracellular tissue tone of adenosine and thereby the degree of adenosine receptor activation (Boison, 2013).

Despite the dynamic changes in cell-type and isoform specific expression patterns of Adk, the transcriptional regulation of the Adk gene remains enigmatic. Here we first characterized and quantified developmental changes in the cell-type and isoform specific generation of Adk transcripts during early postnatal brain development in rat. Coordinated changes in transcription factor binding together with epigenetic mechanisms may play a role in brain development (Bonasio et al., 2010). Therefore, we analyzed transcription factor binding as well as different epigenetic mechanisms including DNA methylation and histone modification at two different developmental time points representing the immature vs. mature adenosine system (P4 and P14) in order to understand their possible implication in the regulation of Adk. We found striking developmental changes in isoform specific Adk transcription, characterized by developmental downregulation of nuclear Adk-L transcripts in neurons and upregulation of cytoplasmic Adk-S transcripts in astrocytes. These coordinated developmental changes in Adk gene transcription were in part facilitated through transcription factor Specificity protein 1 (SP1), but according to our data not based on epigenetic mechanisms.

## Materials and methods

### Animals

Wild-type 4 and 14 day old male and female Wistar rats (Charles River, Sulzfeld, Germany) were used. All rats in the present study were bred and maintained at the local animal center in accordance with the guidelines of the European Federation for Laboratory Animal Science Associations (Directive 2010/63/EU) and with the German Animal Welfare Act. The study was approved by the local animal care and use committee (TS 1/13). Animals were housed in breeding cages under controlled environmental conditions (12 h light/dark cycle, 20–23°C, 50% relative humidity, drinking and feeding *ad libitum*).

### Preparation of cell suspensions and individual cell populations

Rats were anesthetized with isoflurane (CP-Pharma, Burgdorf, Germany), decapitated, and skulls opened. Whole brain was removed and placed into a cold petri dish containing hippocampus dissection buffer (HDB; 6 mg/ml glucose, 10 mM saccharose, 25 mM HEPES, and 5 mM NaHCO3 in HBSS [w/o MgCl2, CaCl2; Life Technologies, Darmstadt, Germany]) until preparation. Preparation of cell suspensions was performed as described elsewhere with modifications (Yang et al., 2010). In brief, hippocampi of each hemisphere were resected and placed into a cold HDB containing petri dish. Hippocampi were washed 3 times with HDB, then transferred into an enzymatic tissue dissociation solution containing 0.01% DNase I, 0.1% dispase II, 0.01% papain (all Roche, Mannheim, Germany) and 12.5 mM MgSO4 in HBSS and mechanically dissociated using a brain specific program of the gentleMACS Octo Dissociator (Miltenyi Biotec, Bergisch Gladbach, Germany). Afterwards the tissue was incubated at 37°C for 30 min, followed by another mechanical dissociation step with the gentleMACS Octo Dissociator and centrifugation at 800 rpm for 5 min at room temperature. The suspension was gently triturated 5 times in serum-free Neurobasal-A Medium (Life Technologies) supplemented with 2% B27 (Life technologies) and passed through a sterile nylon strainer (70 μm). HDB containing 4% bovine serum albumin (BSA; Amresco, Solon, USA) was added and the suspension was centrifuged at 1100 rpm for 8 min at room temperature to remove cell debris. Cell suspensions were then pre-plated onto an uncoated flask and incubated at 37°C in 5% CO_2_ for 1 h. During this time glial cells settled down and adhered to the bottom of the flask, while neurons remained in the supernatant. After pre-plating, the supernatant was collected and centrifuged at 800 rpm for 8 min at room temperature. For gene expression analyses glial cells were detached from the flask and centrifuged at 800 rpm for 8 min at room temperature. Cell pellets were used for subsequent processing.

### Gene expression analysis

Hippocampi from 6 to 7 rat pups were pooled, neuronal and glial cells isolated, and total RNA extracted from each cell pellet using TRIzol Reagent (Life Technologies) according to the manufacturer's instructions with the modification to completely dry the pellet at 56°C for 1 h, and followed by DNase treatment (Life Technologies) to avoid contamination with genomic DNA. First-strand cDNA synthesis was performed using the SuperScript II Reverse Transcriptase Kit (Life Technologies) according to the manufacturer's instructions. Quantitative real-time PCR was performed using the 7500 Fast Real-Time PCR System (Life Technologies) with Power SYBR Green PCR Master Mix (Life Technologies) as fluorescent dye according to the manufacturer's protocol. The following primers were used to selectively amplify each ADK isoform and both isoforms at the same time: AdkRT_1 fw- CCAGAAGCGCTGAGTGAAAAT, rev- GTCTTCGGCCAAGATCTGGT; AdkRT_2 fw- ATGACGTCCACCAGTGAAAAT, rev- GTCTTCGGCCAAG ATCTGGT; AdkRT_3 fw- GTGGCAACCGGTCTCTTGTT, rev- AAACTCTGGCTTTCTCTACCAA; GAPDH fw- GGCTGGCATTGCTCTCAATG, rev- CATGTAGGCCATGAGGTCCA. GAPDH quantification was used as internal reference gene for normalization. Fold differences of mRNA levels were calculated using the ΔΔCt method. No-template controls for each primer were included on every plate and melt curve analysis was performed to exclude unspecific amplification. All PCR reactions were repeated three independent times (i.e., 3 technical replicates) from three different cell preparations (i.e., 3 biological replicates).

### Chromatin immunoprecipitation assay

For ChIP experiments hippocampi of rat pups derived from one litter were used for preparation and cell suspensions were pooled for fixation. Chromatin immunoprecipitation was performed according to the X-ChIP protocol from Abcam with some modifications. In detail, neuronal cell pellets were cross-linked in 0.8% formaldehyde for 10 min at room temperature. The crosslinking reaction was stopped by adding glycine to a final concentration of 0.125 M and centrifuged at 1000 rpm for 8 min at 4°C. Pellet was washed twice in cold PBS and snap frozen in liquid nitrogen. Cells were lysed in SDS-lysis buffer (50 mM Tris-HCl [pH 8.0], 10 mM EDTA dihydrate, 1% SDS) for 1 h and lysis was facilitated by passing the lysate through a 0.4 mm needle several times. The lysate was sheared using a Diagenode Bioruptor on high power for 4 × 5 cycles (30 s on-off). Equal amounts of chromatin lysate (2 μg) were diluted 10 times with RIPA buffer (50 mM Tris-HCl [pH8:0], 150 mM NaCl, 2 mM EDTA dihydrate [pH8.0], 1% NP-40, 0.5% sodium deoxycholate, 0.1% SDS) and pre-cleared with Dynabeads Protein G magnetic beads (Life Technologies) for 1 h. Immunoprecipitation was performed overnight with the corresponding primary IgG antibody along with the Dynabeads Protein G magnetic beads. Immune complexes were washed three times with ChIP wash buffer (0.1% SDS, 1% Triton X-100, 2 mM EDTA dihydrate [pH8.0], 150 mM NaCl, 20 mM Tris-HCl [pH8.0]), once with ChIP final wash buffer (0.1% SDS, 1% Triton X-100, 2 mM EDTA dihydrate [pH8.0], 500 mM NaCl, 20 mM Tris-HCl [pH8.0]) and were then eluted from the beads with 120 μl elution buffer (1% SDS, 0,1 M NaHCO3, pH9.0). Protein-DNA cross-links were reverted by overnight incubation at 65°C and DNA was extracted using the QIAquick PCR Purification Kit (Qiagen) according to the manufacturer's protocol. DNA quantification was done by quantitative real-time PCR using the same protocol as mentioned above. The following antibodies were used: 4 μg H3K9ac (ab10812, abcam, Cambridge, UK), 4 μg H3K4me3 (07–473, Millipore, Molsheim, France), 10 μg H4ac (06–866, Millipore), 10 μl H3S10phK14ac (07–081, Millipore), 4 μg H3K27me3 (07–449, Millipore), 8 μg SP1 (17–601, Millipore) and IgG (Millipore). Primers used for amplification of immunoprecipitated DNA are summarized in Table 1. Hist1H4B was used as control primer for normalization for H3K9ac, H3K4me3, H3S10phK14ac; MyoD for H3K27me3 and DHFR for SP1. All steps were performed at 4°C up to and including the immunoprecipitation step. PBS, SDS-Lysis buffer and RIPA buffer were supplemented with protease inhibitors (EDTA-free protease inhibitor cocktail tablets; Roche) and HDAC inhibitors (sodium butyrate; Calbiochem, Darmstadt, Germany) immediately before application. Nonspecific IgG antibody was included in each ChIP as a negative control to exclude unspecific interaction. All ChIP experiments were repeated three independent times (i.e., 3 technical replicates) for each antibody out of 3 different cells pools (i.e., 3 biological replicates). Subsequent qPCR was also performed in triplicates.

**Table 1:** **Genomic DNA primers used for quantitative real-time PCR to amplify immunoprecipitated DNA**.

**PrimerID**	**Forward primer**	**Reverse primer**
Adk1a	AAACCTGGGGGCTGACAAGTT	ACACCCGGGCTTCCTAGTCA
Adk1b	TCTGTTCCCCGGCTTTTGAGAT	CCTCGGAGGCTGCTAGTCA
Adk1c	GGTCAGCGGGACTAGAAAGA	AAGGGACCCAAAACGCAGTTA
Adk1d	CAGACGCGCGTAGAGCCAAT	TCACCGCGGTTTGGCTCTGC
Adk1e	GCCAAACCGCGGTGAGAGT	CCCACGAACGCAGTCACCAT
Adk1f	TGGTCCGGGGGCTGAGTCTT	GGGCGCCTTCCCGCTAAATA
Adk2a	TGAGTGAGGCCCTCATACTCA	ACATCCAGCGGCATTCATTTAC
Adk2b	CAGCTGTTGCCACGCTTATAGT	CAACCGGGTTCGCAGTCACA
Adk2c	TGAGGCGGGCACTCAACCTT	CACGCGGGCTAGCTGACCA
Adk2d	GCGCTCTCCGTCGCTGAGT	GGCCGCCCTCTGCTTACCT
Adk2e	GCGGGACCGGTAGGTGAAGT	AGGGGGAACGTCCGACAGAA
Adk2f	TGGCCTTGCCCACTTTATTTAG	CTCATGGCCCCAAATTACTTC
Hist1H4B	CGGCCTCATCTACGAGGAGA	CTTAGCCGCCGAAGCCATAGA
MyoD	GGCTGTCCACCCCAGTTTGAATA	GACGAAGTGGGCAAGAGACAGT
Dhfr	CGCGCGGCCGCACTTCCT	GGCCCGCCGCGCATCCTA

### Bisulfite sequencing

Genomic DNA was extracted from neuronal cell pellets using the QIAamp DNA Micro Kit (Qiagen, Hilden, Germany), followed by bisulfite conversion of 1 μg of genomic DNA using the EpiTect Bisulfite Kit (Qiagen) according to the manufacturer's protocol. Regions of interest were amplified using the TaKaRa EpiTaq HS Kit (TaKaRa Clontech, Otsu, Japan) and cloned using the TOPO TA Cloning Kit (Life Technologies) doing blue/white screening. White colonies were selected and grown in LB medium overnight. The plasmid was purified using the GeneJET Plasmid Miniprep Kit (Thermo Scientific, Darmstadt, Germany) and clones were sequenced by the Sanger sequencing method (GATC Biotech, Cologne, Germany). Sequences were quality controlled and aligned using the CLC sequence viewer (CLC bio). The following primers were used to amplify bisulfite converted DNA at the promoter region of the first ADK isoform: AdkBB_1.1 fw AGGGATATTTTTGGAGTTTTAGAGAG, rev-TCAAAAAATACCAATCTCCCTATTC; AdkBB_1.2 fw-AATAGGGAGATTGGTATTTTTTGAG, rev-TTCCACCTTAAACTTCTTAAACTTC; AdkBB_1.3 fw-AGTTTAAGAAGTTTAAGGTGGAAG, rev- AAAATCCAAAACCAAAAAACACTAA, and at the promoter region of the second isoform: AdkBB_2.1 fw-AGTTTGAGGTTTTTTAGGTGGTTG, rev ATCCTAATAACCACCTCCCCTTAC; AdkBB_2.2 fw GTTAGTTTTTTTGTTGGTTAGTTAGTT, rev CAATAATTCTCCCTAAATCTACAAACTC.

### Luciferase assay

A ~500 bp fragment of the rat Adk-L minimal promoter including transcription start and ATG triplet (start codon) and an analog fragment with mutated SP1 binding sites, both flanked by restriction sites for Nhe1 and EcoRV (oligo synthesized by Eurofins Genomics, Ebersberg, Germany), were digested with respective restriction endonucleases (both New England Biolabs, Frankfurt am Main, Germany) and cloned into the pGL4.10 *Firefly* Luciferase vector (Promega, Mannheim, Germany) to produce recombinant constructs (pGL4.Adk-500 and pGL4.Adk-500mut). A construct derived from the pGL4.10 vector containing the Herpes Simplex Virus Thymidine kinase (HSV-TK) minimal promoter in front of the *Firefly* Luciferase gene served as positive control (pGL4.TK). The pRL-TK Vector (Promega) encoding the *Renilla* Luciferase under control of the HSV-TK promoter was used as transfection control. pGL4.Adk-500 as well as pGL4.Adk-500mut vectors were each co-transfected with the pGL4.TK reporter vector into HEK293 cells, with control plasmids encoding *Renilla* Luciferase, using Lipofectamine Transfection Reagent (Thermo Fisher Scientific, Schwerte, Germany). 24 h following transfection HEK cells were incubated for 3 h with different concentrations of recombinant SP1 protein (Sigma-Aldrich, Taufkirchen, Germany) in the growth medium. Luciferase activities were assayed using the Dual-Luciferase Reporter Assay (Promega) according to the manufacturer's protocol. Thereby *Firefly* Luciferase measurements were normalized to *Renilla* Luciferase activity. All Luciferase assays were performed in triplicate.

### Statistical analysis

Statistical analysis was performed using SPSS for Windows version 17.0 and GraphPad Prism6. Differences in gene expression and DNA methylation were compared using Mann-Whitney test, whereas differences in transcription factor binding and chromatin modifications were analyzed using Wilcoxon matched-pairs test. Statistical significance of individual data pairs was further determined using multiple *t*-test comparisons, one per row (i.e., Holm-Sidak method). Data from reporter gene assays was analyzed using Friedman statistic. A p value < 0.05 was considered significant. All data is presented as mean ± standard deviation.

## Results

### Dynamic shifts of Adk gene transcription during early postnatal brain development

**Figure 1:**
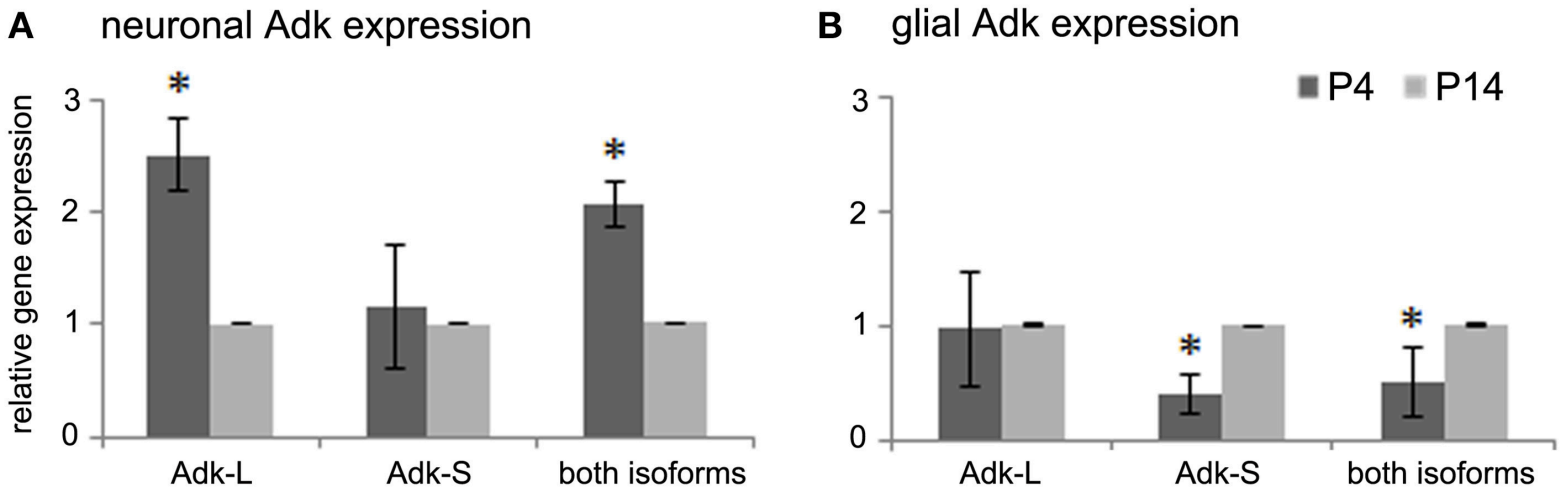
**Adk gene expression in early postnatal brain development. (A)** Adk gene expression of long and short Adk isoforms separately as well as total Adk expression in rat hippocampal neurons and **(B)** in rat hippocampal glial cells at postnatal days 4 and 14 (P4 and P14, respectively) is shown. Adk gene expression was calculated relative to internal reference gene GAPDH. Neuronal and glial Adk gene expression at P4 was normalized to respective expression at P14, which was always set at 100%. Adk-L - long Adk isoform; Adk-S - short Adk isoform. ^*^indicates significance.

To understand the molecular basis for dynamic changes in Adk protein expression during early postnatal brain development we quantified Adk gene expression at early developmental time points in the rat. For experimental procedure see Supplement Figure 1. To quantify cell-type and isoform specific changes in Adk gene transcription we isolated hippocampal neuronal and glial cell populations from rat pups at postnatal days four (P4) and 14 (P14) for quantitative real-time PCR analysis of the long and short isoforms Adk-L and Adk-S. In hippocampal neurons we found a 2.5-fold decrease in gene expression of the long Adk isoform between P4 and P14 (*p* < 0.05), whereas the short isoform was not affected (Figure 1A). In contrast, in glial cells, mRNA levels of the short isoform significantly increased between P4 and P14 (Figure 1B), whereas gene expression of the long Adk isoform was unaffected. These data demonstrate opposing directions of the regulation of the two isoforms of Adk in neurons vs. glia suggesting complex regulatory mechanisms of Adk gene expression.

### Impact of histone modifications on Adk gene expression

**Figure 2:**
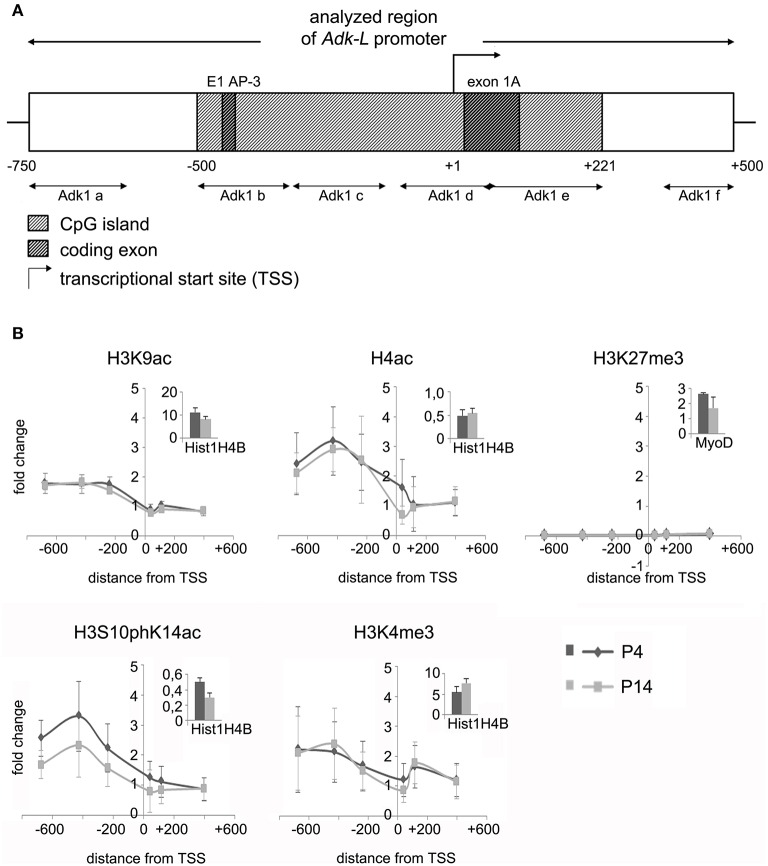
**Impact of epigenetic chromatin modifications on Adk-L promoter activity in P4 and P14 rat hippocampal neurons. (A)** Promoter region of the long Adk isoform was analyzed from pos. −735 to + 470 relative to the TSS. **(B)** Activating histone modifications were identified at the Adk-L promoter in both P4 and P14 hippocampal neurons, including acetylation of H3 and H4, as well as H3 phosphoacetylation and lysine 4 trimethylation, with a relative sparing of the TSS. There was no evidence for repressive H3K27 trimethylation. Upper right corner of each diagram with insets showing enrichment of positive controls for each antibody presented as percent of the total input chromatin (% input). Adk-L promoter, promoter regulating long Adk isoform expression; ChIP, chromatin immunoprecipitation; H3K9ac, acetylation of lysine (K) 9 of histone H3; H4ac, pan-acetylation of histone H4; H3S10phK14ac, phosphoacetylation of histone H3 targeting Serine (S) 10 and Lysine (K) 14; H3K4me3, trimethylation of lysine (K) 4 of histone H3; H3K27me3, trimethylation of lysine (K) 27 of histone H3; TSS, transcriptional start site.

**Figure 3:**
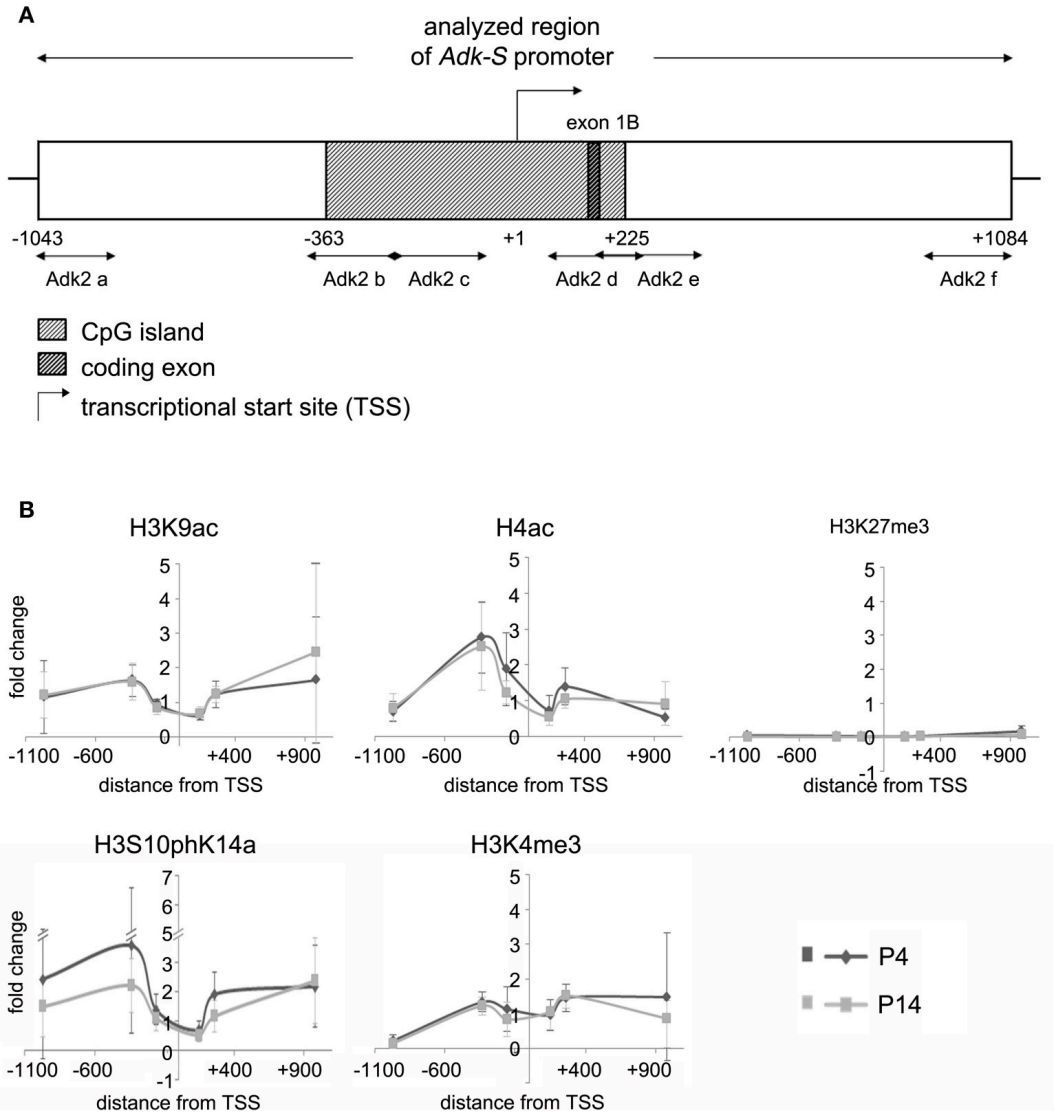
**Impact of epigenetic chromatin modifications on Adk-S promoter activity in P4 and P14 rat hippocampal neurons. (A)** Promoter region of the short Adk isoform was analyzed from pos. −1043 to +1084 relative to the TSS. **(B)** Enrichment of activating histone modifications at the Adk-S promoter in both P4 and P14 hippocampal neurons, including acetylation of H3 and H4, as well as H3 phosphoacetylation and lysine 4 trimethylation, particularly upstream of the TSS was identified. No evidence for repressive H3K27 trimethylation. Adk-S promoter, promoter regulating short Adk isoform expression; ChIP, chromatin immunoprecipitation; H3K9ac, acetylation of lysine (K) 9 of histone H3; H4ac, pan-acetylation of histone H4; H3S10phK14ac, phosphoacetylation of histone H3 targeting Serine (S) 10 and Lysine (K) 14; H3K4me3, trimethylation of lysine (K) 4 of histone H3; H3K27me3, trimethylation of lysine (K) 27 of histone H3; Hist1H4B, Histone cluster 1, H4B; MyoD, Myogenic differentiation 1; TSS, transcriptional start site.

We asked, whether chromatin modifications may play a role in the regulation of Adk isoform expression. Chromatin immunoprecipitation was carried out to evaluate different modifications of histone H3 and H4 at the two promoter regions of long and short Adk isoforms, respectively, using P4 and P14 rat hippocampal neurons. Antibodies directed against acetylated histone H4 and H3 (H4Ac, H3K9Ac), phosphoacetylated H3 (H3S10phK14Ac), and trimethylation of lysine 4 or 27 of histone H3 (H3K4me3, H3K27me3) were applied. Following the enrichment step quantitative real-time PCR with Adk-specific primers was performed to prove binding of histones with respective modifications to the Adk promoters. The amplified regions cover large parts of each promoter region (Adk-L: −735 bp to +470 bp; Adk-S: −1043 bp to +1084 bp) as depicted in Figures 2A, 3A.

We identified histone acetylation of histone H3 and H4 as well as phosphoacetylation at histone H3 at the promoter regions of both Adk isoforms in P4 and in P14 rat hippocampal neurons, especially upstream to the TSS (Figures 2B, 3B). Additionally there was trimethylation of lysine 4 of histone H3 at both Adk promoters. Trimethylation of lysine 27 of histone H3 on the other hand was neither found at the promoter of the long isoform nor at the promoter of the short isoform. In summary, we identified similar compositions of histone modifications at the promoter of the long isoform as at the predicted promoter of the short isoform in both P4 and P14 rat hippocampal neurons.

### Methylation status of Adk promoters in early postnatal brain development

We next focused on promoter DNA methylation. The EMBOSS Cpgplot tool was used for CpG island prediction. Two consecutive CpG islands were identified around the TSS of the long Adk isoform enclosing a region of 755 bp. Furthermore, a single CpG island of 588 bp was predicted to be located within the presumed promoter of the short Adk isoform (Figures 2A, 3A).

**Figure 4:**
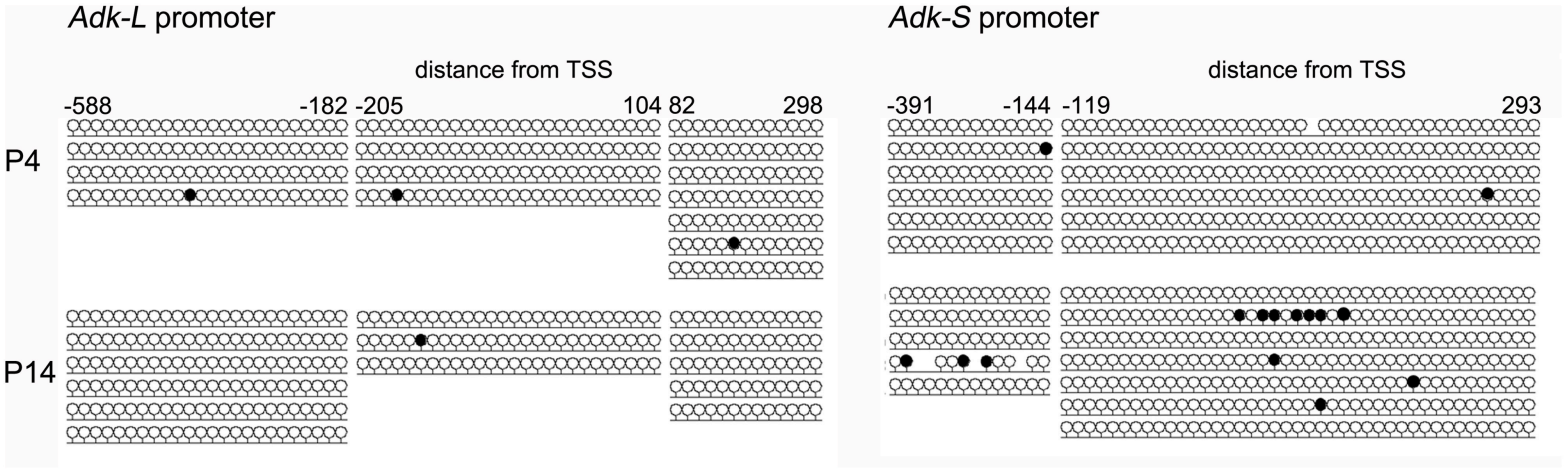
**DNA promoter methylation**. CpG methylation was analyzed using bisulfite sequencing following amplification and subcloning of the region of interest. A minimum of three clones was analyzed per sample. Promoter regions were amplified from pos. −588 to +298 (long isoform) and pos. −391 to +293 (short isoform) relative to TSS.

Harvested DNA from P4 and P14 rat hippocampal neurons was used for amplification (subsequent to bisulfite treatment) and sequencing of the rat Adk promoters including 5′ untranslated region (UTR) and first exon of long (Pos. −588 to +298) and short isoforms (Pos. −391 to +293), respectively. However, both promoters were unmethylated in P4 as well as in P14 rat hippocampal neurons (Figure 4).

### Regulation of Adk expression through transcription factor SP1

**Figure 5:**
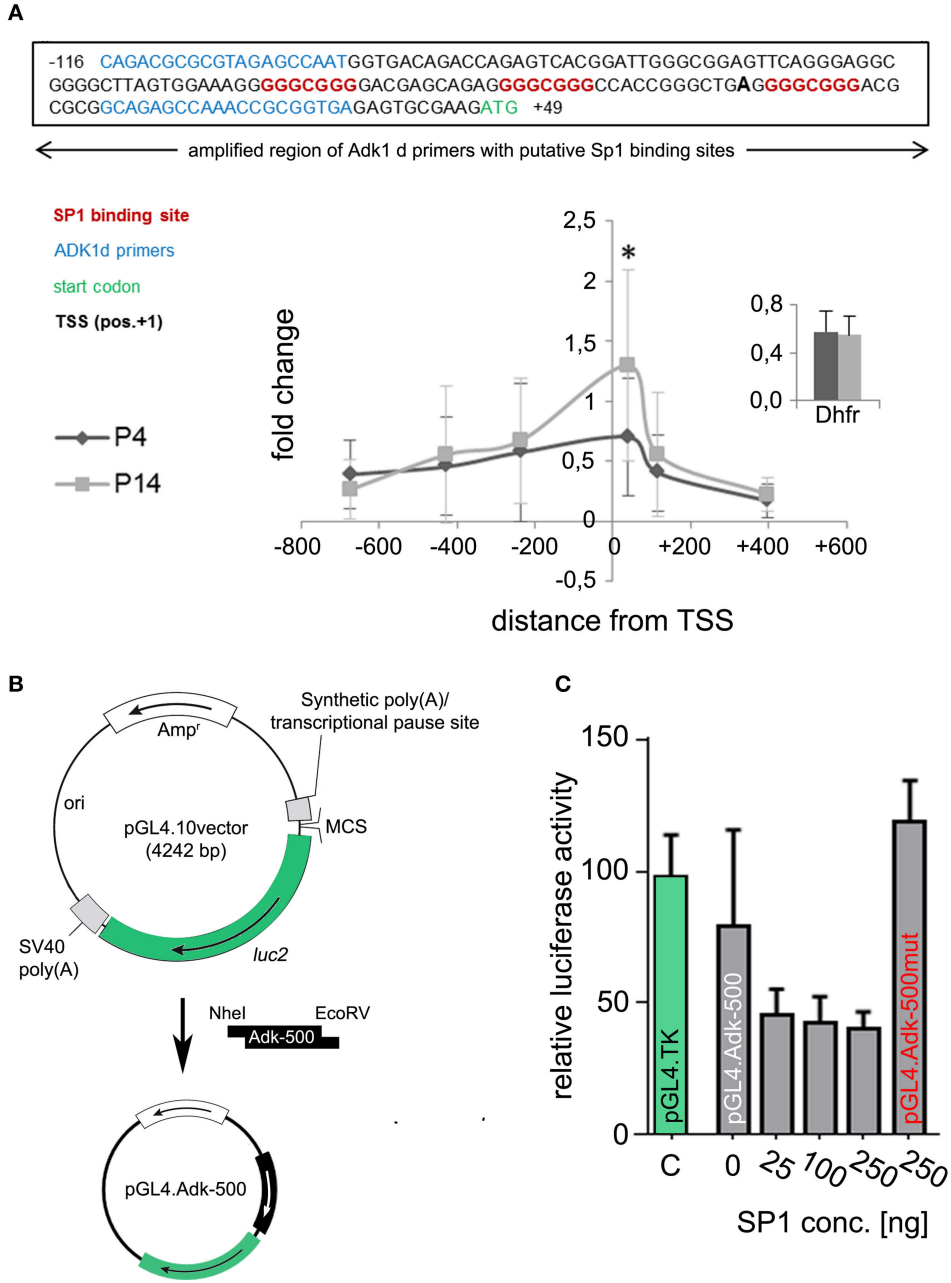
**Identification of transcription factor binding. (A)** Upper panel: SP1 binding sites (indicated in red) at the amplified region of Adk 1d primers (indicated in blue) around the TSS (pos. +1, bold). Lower panel: Binding of SP1 to the promoter region of the long Adk isoform are shown relative to a positive control primer in P4 and P14 rat hippocampal neurons. The analyzed promoter region spanned over 1 kb between position −735 and +470 relative to the TSS. There is significant enrichment of SP1 binding around the TSS of the Adk-L promoter in P14 rat hippocampal neurons, which may be implicated in the suppression of the long Adk isoform. Inset displays enrichment of positive control for Sp1 antibody presented as percent of the total input chromatin (% input). **(B)** Luciferase assay was performed to analyze the binding of SP1 to the Adk promoter and its impact on gene expression. Therefore, the Adk-L promoter including 1.000 bp upstream the TSS was subcloned in front of luciferase 2 (luc2) gene in the pGL4.10 vector and transfected into HEK293 cells. Transfected cells were incubated with increasing concentrations of recombinant SP1 for 3 h and then used for luciferase activity measurements. **(C)** We identified a concentration dependent negative regulation of the Adk-L promoter and luc2 gene through transcription factor SP1. Adk, Adenosine kinase; Adk-L promoter, promoter regulating expression of the long Adk isoform; Ap3m1, adaptor-related protein complex 3, mu 1 subunit; ChIP, chromatin immunoprecipitation; CpG, cytosine guanine dinucleotide; DHFR, Dihydrofolate reductase; kb, kilobase; SP1, specificity protein 1;TSS, transcriptional start site. ^*^indicates significance.

*In silico* analysis of the two alternative Adk gene promoters, which are anticipated to regulate Adk isoform expression, identified CG boxes close to the transcription start for the long, but not the short rat Adk isoform (Figure 5). These elements can be bound by specificity protein 1 (SP1; Figure 5A upper panel). In order to analyze SP1 binding to the Adk-L promoter, chromatin immunoprecipitation was performed in P4 and P14 rat hippocampal neurons. We identified SP1 binding in both P4 and P14 rat hippocampal neurons around the transcription start site (TSS; Figure 5A lower panel). Wilcoxon rank test identified no significant difference in SP1 binding to the regulatory promoter region of the long Adk isoform between the two groups. Multiple *t*-test analysis for each data pair (P4 vs. P14) along the promoter identified significantly increased SP1 binding at position −102 to +50 relative to the TSS in P14 neurons (Holm-Sidak method, p=0.0098). This region covers the three putative SP1 binding sites (Figure 5A upper panel).

To prove binding of SP1 to the Adk-L promoter and thus SP1-mediated suppression of gene expression we transiently transfected HEK293 cells with reporter plasmids containing Luciferase under the control of the minimal Adk-L promoter covering ~500 bp around the TSS including all three SP1 binding sites (Adk-500; Figure 5B). Reporter plasmids containing the Herpes Simplex Virus thymidine kinase minimal promoter (HSV-TK) served as positive control. Assessment of Luciferase activity revealed promoter activity for the chosen genomic region (Figure 5C). Promoter activity for the Adk-500 promoter was slightly lower than HSV-TK promoter activity in HEK293 cells. Incubation with increasing concentrations of recombinant SP1 in the culture medium over 3 h reduced promoter activity significantly suggesting concentration dependent regulation of Adk-L promoter activity by SP1 binding (Friedman test, *p* = 0.018, Figure 5C). Upon mutation of all three SP1 binding sites activity of the Adk-L minimal promoter was restored back to control levels and entirely independent of supplemented SP1 (pGL4.Adk-500mut; Figure 5C). Our experimental finding on transcription factor binding validate *in silico* data on the existence of a second active Adk promoter (Cui et al., 2011). Differential binding of SP1 to the Adk-L promoter in hippocampal neurons of P4 and P14 rats seemed to have contributed to the dynamic Adk expression pattern.

## Discussion

Adk is an important neurodevelopmental gene, with broad implications for brain homeostasis and function. Adk-null mice have transmethylation deficits, develop hepatic steatosis, and die during early postnatal development (Boison et al., 2002b). Likewise, ADK-deficient patients have transmethylation deficits and develop hepatic encephalopathy (Bjursell et al., 2011). Conditional ADK mutants suggest a functional link between adenosine homeostasis and higher order brain function (Fedele et al., 2005; Pignataro et al., 2007; Yee et al., 2007; Li T. et al., 2008; Palchykova et al., 2010; Shen et al., 2011; Wu et al., 2013; Diógenes et al., 2014). Moreover, ADK dysfunction is involved in several neurological and neuropsychiatric pathologies whereas corrective reconstruction of adenosine signaling is of therapeutic value (Boison et al., 2002a, 2012; Boison and Aronica, 2015). Given the translational significance of ADK-based regulation of adenosine homeostasis, understanding the mechanisms underlying Adk gene regulation, and hence the regulation of adenosine metabolism, is of practical value for clinical translation. Our findings indicate that Adk gene expression is tightly controlled during early postnatal brain development both in a cell-type and isoform selective manner. Several aspects of our work warrant further discussion.

### Adk gene structure

The Adk gene is one of the largest genes in the mammalian genome (> 350 kb in mice, rats, and humans). Its coding sequence is highly conserved in vertebrates. The rat Adk gene, as all known mammalian Adk genes, consists of 11 short exons and exceptionally large introns. There are two ADK isoforms, long and short (ADK-L and ADK-S), expressed in mammalian cells (Cui et al., 2009). Both isoforms are identical except for the first exon. The first exon of the short isoform is located in the intronic sequence between the first and second exon of the long isoform. Both isoforms are most likely regulated by two independent promoters (Cui et al., 2009). The promoter of the first isoform is a bidirectional promoter regulating also the expression of the clathrin adaptor subunit μ3A gene (Ap3m1) lying upstream of the Adk gene (Cui et al., 2009). Genomic organization and linkage of the Ap3m1 and Adk genes via a bidirectional promoter has been validated by deletion mutants of cultured Chinese hamster cells (Singh and Gupta, 2004) and is conserved between species, e.g., rats, mice and humans (see ENSEMBL genome browser; Yates et al., 2016). However, the promoter of the second isoform was identified only *in silico*, upstream of the first exon of the short isoform (in the intronic region) containing a CpG island and transcription factor binding sites (Cui et al., 2009). Here we provide the first experimental evidence for the existence of a second promoter as we identified specific histone modifications and selective transcription factor binding sites indicative of an active gene promoter.

### Coordinated changes in Adk gene expression

There is strong evidence that temporal and spatial ADK isoform expression is highly regulated. For mammalian brain ADK protein a dramatic switch from neuronal toward glial, and long toward short Adk isoform expression has been described during early postnatal brain development (Studer et al., 2006; Cui et al., 2009). In rodents the described changes occur during the first three postnatal weeks, with major shifts in hippocampal ADK protein expression occurring toward the end of the first postnatal week. These coordinated changes on the protein level suggest the existence of matching regulatory mechanisms on the level of gene transcription. We therefore analyzed Adk gene expression in the rat hippocampus at two distinct developmental stages, an early time-point reflecting largely neuronal ADK expression (P4) and a later time-point reflecting largely astroglial ADK expression (P14). In line with protein data (Studer et al., 2006) we provide evidence that at P4 the Adk gene is predominantly expressed in hippocampal neurons, whereas later at P14 it seemed to be predominantly expressed in glial cells. Strikingly we found opposing shifts in isoform expression in different cell types with downregulation of the nuclear isoform in neurons and upregulation of the cytoplasmic isoform in astrocytes during this developmental time window. These findings suggest the need for specific regulatory mechanisms governing cell-type and isoform selective Adk gene expression. Those findings prompted our subsequent evaluation of mechanisms implicated in the regulation of gene expression.

### Regulation by epigenetic modifications

To further delineate the mechanisms through which the switch in Adk gene expression in the rodent brain is mediated, we analyzed epigenetic chromatin modifications at the Adk-L and Adk-S promoter region in rat hippocampal neurons at two developmental stages that are characterized by stable neuronal (P4) and stable astrocytic expression (P14) of Adk. Epigenetic gene regulation is very complex, which ensures nuclear processes (i.e., transcription, replication, DNA damage response) to be directed to the required region of the genome at appropriate time points mediating unique cellular responses and biological outcomes. This same complexity is what makes analysis and interpretation of epigenetic modifications difficult. However, some signals are more understood than others, e.g., promoters of active genes are commonly associated with acetylated H3K9 (H3K9ac) and H4. Likewise, phosphoacetylation of histone H3 (e.g., H3S10phK14ac) concomitant with gene activation is well established and has been observed at several inducible genes (Clayton and Mahadevan, 2003). The mark H3K4me3 is also found at active gene promoters as well as promoters of genes that are poised for activation. In contrast, temporarily inactive genes, such as in undifferentiated cells are marked by H3K27me3, a mark of facultative heterochromatin, with or without the transcriptionally permissive H3K4me3 (Bernstein et al., 2006). We detected activating histone modifications including H3K9Ac, H3S10phK14Ac, H4Ac, and H3K4me3 at the promoter regions regulating Adk isoform expression in both P4 and P14 rat hippocampal neurons. Prevalence of histone H3 and H4 acetylation as well as H3K4me3 provide a typical signature for CpG island promoters (Guenther et al., 2007; Mikkelsen et al., 2007). In the present study there was no experimental evidence for repressive trimethylation of H3K27. In addition, CpG islands embedded in the two Adk promoters were found to be unmethylated, which is in concordance with previous findings on the regulation of developmental genes (Weber et al., 2007; Borgel et al., 2010; Numata et al., 2012). Taken together, our data point to open chromatin structure within the Adk promoters in both P4 and P14 rat hippocampal neurons. However, our analysis covered only a small minority of all known histone modifications, which is why an influence of the histone code on differential Adk isoform expression during brain development cannot be excluded. Furthermore, we focused on the promoter region, but did not analyze other regulatory elements such as enhancer, silencer or insulator elements. In this context, it should be noted that the Adk-L promoter is a bidirectional promoter, which also controls transcription of a clathrin adaptor-related protein complex subunit. Regulation of Adk expression by intragenic signatures can also be anticipated. Since the Adk gene is an evolutionary highly conserved gene containing extremely large intronic sequences in higher eukaryotes, it is possible that these sequences harbor regulatory elements to control Adk expression. Interestingly, the Adk gene is much smaller in lower eukaryotes suggesting that regulatory elements which are needed for tissue and development-specific regulation in higher eukaryotes are located in growing intronic sequences (Singh et al., 2001; Singh and Gupta, 2004).

### Regulation through transcription factor binding

Expression of eukaryotic genes is primarily controlled at the level of transcription initiation, although in some cases it may be attenuated and regulated at subsequent steps. Promoters contain cis-acting sequences which serve as binding sites for a wide variety of regulatory factors that control the expression of individual genes. Here we performed in silico analysis of the two Adk promoters and identified three putative binding sites for specificity protein 1 (SP1) in close proximity to the transcriptional start site (TSS) of the long Adk isoform. SP1 binds with high affinity to GC-rich motifs and regulates the expression of a large number of genes involved in a variety of processes including cell growth, apoptosis, and differentiation (Wierstra, 2008). Previous Adk promoter studies have shown that deletion of the first 200 bp upstream of the start codon ATG lead to a 3-fold increased promoter activity, suggesting inhibitory elements located in this region (Singh and Gupta, 2004). The significantly increased binding of SP1 to this distinct part of the Adk-L promoter in P14 neurons points to an inhibitory effect of SP1 binding on the expression of the long Adk isoform. In fact, luciferase reporter gene (luc2) under the control of the Adk-L promoter (500 bp upstream the TSS) showed a concentration dependent effect of SP1 binding on luc2 gene expression in HEK cells, which could be completely blocked by mutating SP1 binding sites. However, there is also low, but present binding of SP1 to the Adk-L promoter in rat P4 neurons. Since SP1 can activate and silence gene expression and is highly regulated by post-translational modification, e.g., phosphorylation, sumoylation, proteolytic cleavage, glycosylation, and acetylation (Wierstra, 2008), it might be possible that additional mechanisms beyond SP1 binding itself are involved in the fine tuning of Adk expression.

### Conclusions and outlook

We documented dynamic changes in Adk gene transcription during early postnatal brain development. Two selective time points were investigated, P4 and P14, respectively, flanking the narrow time window when Adk expression changes from the long neuronal to the short glial isoform. We identified altered binding of transcription factor SP1 to the Adk-L promoter in close proximity to the TSS. Despite previous reports linking SP1 binding to downstream epigenetic changes we did not find any alterations with regard to common activating and silencing chromatin marks. Our data explain some mechanisms underlying the temporal and spatial changes in Adk gene expression in the postnatal rodent brain, but for obvious reasons are non-exhaustive. Future studies need to be conducted to further delineate the role of other epigenetic regulatory events and the relevance of the time window investigated as well as the potential implication of other, so far unconsidered, more archaic regulatory mechanism outside epigenetics, e.g., through the availability (excess or lack) of adenosine, to control Adk gene expression. Such archaic mechanisms might be meaningful, because the long Adk primary transcripts (>400,000 residues in the rat) sequester significant amounts of adenosine, suggesting that Adk gene transcription might be regulated by the availability of adenosine, an interesting possibility that warrants further exploration.

## Author contributions

KKo conceived experiments, performed statistical analysis, and prepared figures. KKi and JJ performed experiments and prepared figures. All authors (KKo, KKi, JJ, DB) wrote the manuscript.

## Funding

Our work was supported by the German Research Council (DFG Bl 421/3-1) as part of the European Science Foundation EUROCORES Programme EuroEPINOMICS (EpiGENet; KKi, KKo), by the European Union's Seventh Framework Program (DESIRE project, grant agreement #602531; KKi, KKo, JJ) as well as through NIH grant NS084920 (DB).

### Conflict of interest statement

The authors declare that the research was conducted in the absence of any commercial or financial relationships that could be construed as a potential conflict of interest.

## Supplementary material

Supplement Figure 1 **Brains of P4 and P14 rat pups were removed, hippocampi resected and then enzymatically and mechanically dissociated**. Cell suspension was pre-plated for 1 h, to separate glial (yellow) and neuronal cell populations (gray) by sedimentation. Afterwards, cells were immediately fixed with formalin or lysed for downstream applications.

## References

[B1] Aronica E. Zurolo E. Iyer A. de Groot M. Anink J. Carbonell C.. (2011). Upregulation of adenosine kinase in astrocytes in experimental and human temporal lobe epilepsy. Epilepsia 52, 1645–1655. 10.1111/j.1528-1167.2011.03115.x 21635241 PMC3169746

[B2] Bernstein B. E. Mikkelsen T. S. Xie X. Kamal M. Huebert D. J. Cuff J.. (2006). A bivalent chromatin structure marks key developmental genes in embryonic stem cells. Cell 125, 315–326. 10.1016/j.cell.2006.02.041 16630819

[B3] Bjursell M. K. Blom H. J. Cayuela J. A. Engvall M. L. Lesko N. Wedell A.. (2011). Adenosine kinase deficiency disrupts the methionine cycle and causes hypermethioninemia, encephalopathy, and abnormal liver function. Am. J. Hum. Genet. 89, 507–515. 10.1016/j.ajhg.2011.09.004 21963049 PMC3188832

[B4] Boison D. (2013). Adenosine kinase, exploitation for therapeutic gain. Pharmacol. Rev. 65, 906–943. 10.1124/pr.112.006361 23592612 PMC3698936

[B5] Boison D. Aronica E. (2015). Comorbidities in Neurology, Is adenosine the common link? Neuropharmacology 97, 18–34. 10.1016/j.neuropharm.2015.04.031 25979489 PMC4537378

[B6] Boison D. Huber A. Padrun V. Déglon N. Aebischer P. Mohler H. (2002a). Seizure suppression by adenosine-releasing cells is independent of seizure frequency. Epilepsia 43, 788–796. 10.1046/j.1528-1157.2002.33001.x 12180995

[B7] Boison D. Scheurer L. Zumsteg V. Rülicke T. Litynski P. Fowler B.. (2002b). Neonatal hepatic steatosis by disruption of the adenosine kinase gene. Proc. Natl. Acad. Sci. U.S.A. 99, 6985–6990. 10.1073/pnas.092642899 11997462 PMC124515

[B8] Boison D. Singer P. Shen H. Y. Feldon J. Yee B. K. (2012). Adenosine hypothesis of schizophrenia–opportunities for pharmacotherapy. Neuropharmacology 62, 1527–1543. 10.1016/j.neuropharm.2011.01.048 21315743 PMC3119785

[B9] Bonasio R. Tu S. Reinberg D. (2010). Molecular signals of epigenetic states. Science 330, 612–616. 10.1126/science.1191078 21030644 PMC3772643

[B10] Borgel J. Guibert S. Li Y. Chiba H. Schubeler D. Sasaki H.. (2010). Targets and dynamics of promoter DNA methylation during early mouse development. Nat. Genet. 42, 1093–1100. 10.1038/ng.708 21057502

[B11] Clayton A. L. Mahadevan L. C. (2003). MAP kinase-mediated phosphoacetylation of histone H3 and inducible gene regulation. FEBS Lett. 546, 51–58. 10.1016/S0014-5793(03)00451-4 12829236

[B12] Cui X. A. Agarwal T. Singh B. Gupta R. S. (2011). Molecular characterization of Chinese hamster cells mutants affected in adenosine kinase and showing novel genetic and biochemical characteristics. BMC Biochem. 12:22. 10.1186/1471-2091-12-22 21586167 PMC3118340

[B13] Cui X. A. Singh B. Park J. Gupta R. S. (2009). Subcellular localization of adenosine kinase in mammalian cells, The long isoform of AdK is localized in the nucleus. Biochem. Biophys. Res. Commun. 388, 46–50. 10.1016/j.bbrc.2009.07.106 19635462

[B14] Denessiouk K. A. Rantanen V. V. Johnson M. S. (2001). Adenine recognition, a motif present in ATP-, CoA-, NAD-, NADP-, and FAD-dependent proteins. Proteins 44, 282–291. 10.1002/prot.1093 11455601

[B15] Diógenes M. J. Neves-Tomé R. Fucile S. Martinello K. Scianni M. Theofilas P.. (2014). Homeostatic control of synaptic activity by endogenous adenosine is mediated by adenosine kinase. Cereb. Cortex 24, 67–80. 10.1093/cercor/bhs284 22997174 PMC3862265

[B16] Duarte A. I. Proença T. Oliveira C. R. Santos M. S. Rego A. C. (2006). Insulin restores metabolic function in cultured cortical neurons subjected to oxidative stress. Diabetes 55, 2863–2870. 10.2337/db06-0030 17003354

[B17] Ethier M. F. Dobson J. G. Jr. (1997). Adenosine stimulation of DNA synthesis in human endothelial cells. Am. J. Physiol. 272(3 Pt 2), H1470–H1479. 9087626 10.1152/ajpheart.1997.272.3.H1470

[B18] Fedele D. E. Gouder N. Güttinger M. Gabernet L. Scheurer L. Rulicke T.. (2005). Astrogliosis in epilepsy leads to overexpression of adenosine kinase, resulting in seizure aggravation. Brain 128(Pt 10), 2383–2395. 10.1093/brain/awh555 15930047

[B19] Fredholm B. B. Arslan G. Halldner L. Kull B. Schulte G. Wasserman W. (2000). Structure and function of adenosine receptors and their genes. Naunyn Schmiedebergs. Arch. Pharmacol. 362, 364–374. 10.1007/s002100000313 11111830

[B20] Giglioni S. Leoncini R. Aceto E. Chessa A. Civitelli S. Bernini A.. (2008). Adenosine kinase gene expression in human colorectal cancer. Nucleosides Nucleotides Nucleic Acids 27, 750–754. 10.1080/15257770802145629 18600536

[B21] Guenther M. G. Levine S. S. Boyer L. A. Jaenisch R. Young R. A. (2007). A chromatin landmark and transcription initiation at most promoters in human cells. Cell 130, 77–88. 10.1016/j.cell.2007.05.042 17632057 PMC3200295

[B22] Kobow K. Blumcke I. (2012). The emerging role of DNA methylation in epileptogenesis. Epilepsia 53(Suppl. 9), 11–20. 10.1111/epi.12031 23216575

[B23] Li H. Smolen G. A. Beers L. F. Xia L. Gerald W. Lee S. B.. (2008). Adenosine transporter ENT4 is a direct target of EWS/WT1 translocation product and is highly expressed in desmoplastic small round cell tumor. PLoS ONE 3:e2353. 10.1371/journal.pone.0002353 18523561 PMC2394657

[B24] Li T. Ren G. Lusardi T. Wilz A. Lan J. Q. Iwasato T.. (2008). Adenosine kinase is a target for the prediction and prevention of epileptogenesis in mice. J. Clin. Invest. 118, 571–582. 10.1172/jci33737 18172552 PMC2157568

[B25] MacLaughlin M. Martinez-Salgado C. Eleno N. Olivera A. Lopez-Novoa J. M. (1997). Adenosine activates mesangial cell proliferation. Cell Signal 9, 59–63. 10.1016/S0898-6568(96)00091-5 9067631

[B26] Masino S. A. Li T. Theofilas P. Sandau U. S. Ruskin D. N. Fredholm B. B.. (2011). A ketogenic diet suppresses seizures in mice through adenosine A receptors. J. Clin. Invest. 121, 2679–2683. 10.1172/JCI57813 21701065 PMC3223846

[B27] Mikkelsen T. S. Ku M. Jaffe D. B. Issac B. Lieberman E. Giannoukos G.. (2007). Genome-wide maps of chromatin state in pluripotent and lineage-committed cells. Nature 448, 553–560. 10.1038/nature06008 17603471 PMC2921165

[B28] Numata S. Ye T. Hyde T. M. Guitart-Navarro X. Tao R. Wininger M.. (2012). DNA methylation signatures in development and aging of the human prefrontal cortex. Am. J. Hum. Genet. 90, 260–272. 10.1016/j.ajhg.2011.12.020 22305529 PMC3276664

[B29] Palchykova S. Winsky-Sommerer R. Shen H. Y. Boison D. Gerling A. Tobler I. (2010). Manipulation of adenosine kinase affects sleep regulation in mice. J. Neurosci. 30, 13157–13165. 10.1523/JNEUROSCI.1359-10.2010 20881134 PMC2950004

[B30] Park J. Gupta R. S. (2008). Adenosine kinase and ribokinase–the RK family of proteins. Cell. Mol. Life Sci. 65, 2875–2896. 10.1007/s00018-008-8123-1 18560757 PMC11131688

[B31] Pawelczyk T. Sakowicz M. Szczepanska-Konkel M. Angielski S. (2000). Decreased expression of adenosine kinase in streptozotocin-induced diabetes mellitus rats. Arch. Biochem. Biophys. 375, 1–6. 10.1006/abbi.1999.1548 10683243

[B32] Pignataro G. Simon R. P. Boison D. (2007). Transgenic overexpression of adenosine kinase aggravates cell death in ischemia. J. Cereb. Blood Flow Metab. 27, 1–5. 10.1038/sj.jcbfm.9600334 16685255

[B33] Saitoh M. Nagai K. Nakagawa K. Yamamura T. Yamamoto S. Nishizaki T. (2004). Adenosine induces apoptosis in the human gastric cancer cells via an intrinsic pathway relevant to activation of AMP-activated protein kinase. Biochem. Pharmacol. 67, 2005–2011. 10.1016/j.bcp.2004.01.020 15130776

[B34] Sakowicz-Burkiewicz M. Kocbuch K. Grden M. Szutowicz A. Pawelczyk T. (2006). Diabetes-induced decrease of adenosine kinase expression impairs the proliferation potential of diabetic rat T lymphocytes. Immunology 118, 402–412. 10.1111/j.1365-2567.2006.02380.x 16827901 PMC1782307

[B35] Shen H. Y. Lusardi T. A. Williams-Karnesky R. L. Lan J. Q. Poulsen D. J. Boison D. (2011). Adenosine kinase determines the degree of brain injury after ischemic stroke in mice. J. Cereb. Blood Flow Metab. 31, 1648–1659. 10.1038/jcbfm.2011.30 21427729 PMC3137468

[B36] Singh B. Gupta R. S. (2004). Genomic organization and linkage via a bidirectional promoter of the AP-3 (adaptor protein-3) mu3A and AK (adenosine kinase) genes, deletion mutants of AK in Chinese hamster cells extend into the AP-3 mu3A gene. Biochem. J. 378(Pt 2), 519–528. 10.1042/bj20031219 14575525 PMC1223951

[B37] Singh B. Lin A. Wu Z. C. Gupta R. S. (2001). Gene structure for adenosine kinase in Chinese hamster and human: high-frequency mutants of CHO cells involve deletions of several introns and exons. DNA Cell Biol. 20, 53–65. 10.1089/10445490150504693 11242543

[B38] Studer F. E. Fedele D. E. Marowsky A. Schwerdel C. Wernli K. Vogt K.. (2006). Shift of adenosine kinase expression from neurons to astrocytes during postnatal development suggests dual functionality of the enzyme. Neuroscience 142, 125–137. 10.1016/j.neuroscience.2006.06.016 16859834

[B39] Tsuchiya A. Kanno T. Saito M. Miyoshi Y. Gotoh A. Nakano T.. (2012). Intracellularly transported adenosine induces apoptosis in [corrected] MCF-7 human breast cancer cells by accumulating AMID in the nucleus. Cancer Lett. 321, 65–72. 10.1016/j.canlet.2012.02.023 22388174

[B40] Weber M. Hellmann I. Stadler M. B. Ramos L. Paabo S. Rebhan M.. (2007). Distribution, silencing potential and evolutionary impact of promoter DNA methylation in the human genome. Nat. Genet. 39, 457–466. 10.1038/ng1990 17334365

[B41] Wierstra I. (2008). Sp1: Emerging roles—Beyond constitutive activation of TATA-less housekeeping genes. Biochem. Biophys. Res. Commun. 372, 1–13. 10.1016/j.bbrc.2008.03.074 18364237

[B42] Williams-Karnesky R. L. Sandau U. S. Lusardi T. A. Lytle N. K. Farrell J. M. Pritchard E. M.. (2013). Epigenetic changes induced by adenosine augmentation therapy prevent epileptogenesis. J. Clin. Invest. 123, 3552–3563. 10.1172/JCI65636 23863710 PMC3726154

[B43] Wu M. Sahbaie P. Zheng M. Lobato R. Boison D. Clark J.. (2013). Opiate-induced changes in brain adenosine levels and narcotic drug responses. Neuroscience 228, 235–242. 10.1016/j.neuroscience.2012.10.031 23098802 PMC3525713

[B44] Yang H. Cong R. Na L. Ju G. You S.-W. (2010). Long-term primary culture of highly-pure rat embryonic hippocampal neurons of low-density. Neurochem. Res. 35, 1333–1342. 10.1007/s11064-010-0189-0 20503070

[B45] Yates A. Akanni W. Amode M. R. Barrell D. Billis K. Flicek P.. (2016). Ensembl 2016. Nucleic Acids Res. 44, D710–D716. 10.1093/nar/gkv1157 26687719 PMC4702834

[B46] Yee B. K. Singer P. Chen J. F. Feldon J. Boison D. (2007). Transgenic overexpression of adenosine kinase in brain leads to multiple learning impairments and altered sensitivity to psychomimetic drugs. Eur. J. Neurosci. 26, 3237–3252. 10.1111/j.1460-9568.2007.05897.x 18005073

