# Supplementary figures and images for: Dynamic Regulation of the Adenosine Kinase Gene during Early Postnatal Brain Development and Maturation
Source: Front Mol Neurosci. 2016 Oct 20;9:99. doi: 10.3389/fnmol.2016.00099 (PMC5071315; doi:10.3389/fnmol.2016.00099)

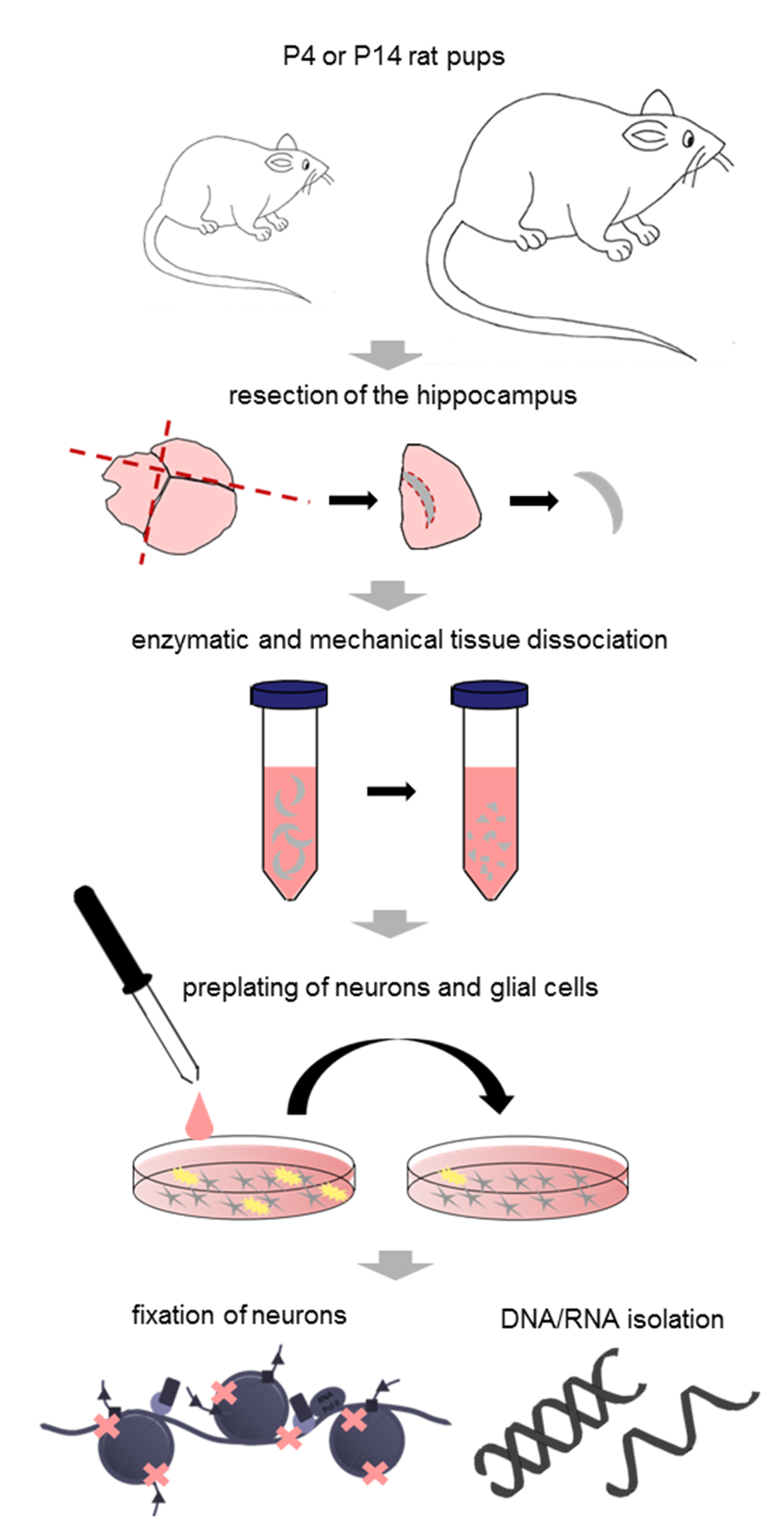

Supplement: Supplement Figure 1 — Brains of P4 and P14 rat pups were removed, hippocampi resected and then enzymatically and mechanically dissociated. Cell suspension was pre-plated for 1 h, to separate glial (yellow) and neuronal cell populations (gray) by sedimentation. Afterwards, cells were immediately fixed with formalin or lysed for downstream applications. [file Image1.TIF]
